# Emerging Roles of Vitamin B_12_ in Aging and Inflammation

**DOI:** 10.3390/ijms25095044

**Published:** 2024-05-06

**Authors:** Sergey Yu. Simonenko, Daria A. Bogdanova, Nikita A. Kuldyushev

**Affiliations:** 1 Research Center for Translational Medicine, Sirius University of Science and Technology, 354340 Sochi, Russia; simonenko.sy@talantiuspeh.ru; 2 Division of Immunobiology and Biomedicine, Center for Genetics and Life Sciences, Sirius University of Science and Technology, 354340 Sochi, Russia

**Keywords:** vitamin B_12_, cobalamin, senescence, aging, protein modifications, inflammation

## Abstract

Vitamin B_12_ (cobalamin) is an essential nutrient for humans and animals. Metabolically active forms of B_12_-methylcobalamin and 5-deoxyadenosylcobalamin are cofactors for the enzymes methionine synthase and mitochondrial methylmalonyl-CoA mutase. Malfunction of these enzymes due to a scarcity of vitamin B_12_ leads to disturbance of one-carbon metabolism and impaired mitochondrial function. A significant fraction of the population (up to 20%) is deficient in vitamin B_12_, with a higher rate of deficiency among elderly people. B_12_ deficiency is associated with numerous hallmarks of aging at the cellular and organismal levels. Cellular senescence is characterized by high levels of DNA damage by metabolic abnormalities, increased mitochondrial dysfunction, and disturbance of epigenetic regulation. B_12_ deficiency could be responsible for or play a crucial part in these disorders. In this review, we focus on a comprehensive analysis of molecular mechanisms through which vitamin B_12_ influences aging. We review new data about how deficiency in vitamin B_12_ may accelerate cellular aging. Despite indications that vitamin B_12_ has an important role in health and healthy aging, knowledge of the influence of vitamin B_12_ on aging is still limited and requires further research.

## 1. Introduction

Vitamin B_12_ is the generic name for a group of cobalamins that play an important role in physiological processes. Recent studies elucidate that vitamin B_12_ may be involved in several pathological processes: change in methylation of DNA [1], aberrant protein post-translational modifications [2], change in microbial composition in the gut [3], change in tumorigenesis probability [4], and modulation of inflammation [5]. We discuss how these B_12_-associated processes may contribute to acceleration of aging and senescence and to inflammation. Apart from the increased risk of age-related diseases, such as cognitive decline, cardiovascular diseases, osteoporosis, and oxidative stress, which are alleviated by supplementation of vitamin B_12_ [6,7], we propose that vitamin B_12_ may play a role in the stimulation of cellular senescence and the pro-inflammatory senescence-associated secretory phenotype through modulation of microbiota, mitochondrial damage, cryptic transcription, and inflammaging (chronic low-grade inflammation in elderly individuals).

**Figure 1:**
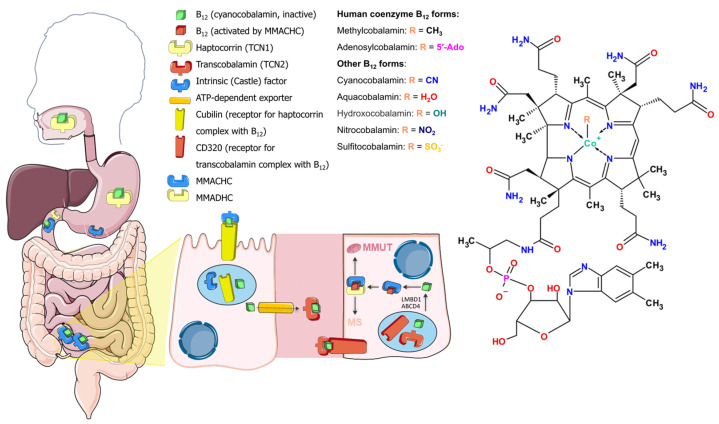
Vitamin B_12_ transport and cobalamin structural forms.

Cobalamins consist of an almost planar corrinoid core with a cobalt atom in the center and two ligands orthogonal to the corrinoid plane. The (“lower”) alpha-ligand is 5′,6′-dimethylbenzimidazole, while the “upper” beta-ligands are different in the different forms of cobalamin (Figure 1). The chemical synthesis of vitamin B_12_ is cumbersome because it includes over 60 steps and provides a 1% yield [8]. Biosynthesis of natural cobalamins is restricted to only few prokaryotes, including human gut residents such as *Pseudomonas*, *Propionibacterium*, *Bacteroides*, *Akkermansia*, and others [9,10,11]. Humans cannot synthesize vitamin B_12_ de novo and need to ingest it from animal-based food [9]. Cyanocobalamin is a metabolically inactive vitamin B_12_ form produced by industrial bacterial strains and is cyanodified during production to improve air stability (Figure 1) [12]. Metabolically active forms of vitamin B_12_ in mammals are methylcobalamin (MeCbl) and 5-deoxyadenosylcobalamin (coenzyme B_12_, AdoCbl) (Figure 1). The former is a cofactor for the enzyme methionine synthase, which is active in the cytosol, while the latter is a cofactor for the enzyme methylmalonyl-CoA mutase in mitochondria.

## 2. Vitamin B_12_ Transport

On the way to its destination, vitamin B_12_ forms complexes with several proteins. Orally ingested B_12_ binds to a protein, haptocorrin, in the upper part of the digestive system, where cobalamin is released from digested proteins by the acidic environment and proteases (Figure 1). The Cbl–haptocorrin complex protects Cbl from this aggressive environment. In the duodenum, haptocorrin is cleaved by pancreatic proteolytic enzymes, liberating cobalamin from the complex. Vitamin B_12_ then binds to the intrinsic factor, which discriminates it from other corrinoids [13]. In the distal ileum, enterocytes uptake vitamin B_12_ only in complex with the intrinsic factor. Exocytosed from enterocytes to blood, free vitamin B_12_ mostly binds to haptocorrin, and a lesser fraction of vitamin B_12_ binds to transcobalamin [14]. Cells absorb vitamin B_12_ in complex with transcobalamin, so-called holotranscobalamin, by a transcobalamin receptor, CD320 (Figure 1) [15]. All RNAs for the transcobalamin receptor subunits and transcobalamin are translated by specific ribosomes (denoted as RPS25/eS25) [16]. Renal re-absorption is accomplished by uptake of holotranscobalamin with another B_12_ receptor, megalin [17]. After endocytosis, transcobalamin releases vitamin B_12_ in lysosomal acidic pH, and free vitamin B_12_ is transported to the cytosol via ATP-dependent transport by lipocalin-1 interacting membrane receptor domain containing protein 1 (LMBD1) and adenosine triphosphate (ATP)-binding cassette subfamily D member 4 (ABCD4) [18,19]. Within the cytosol, vitamin B_12_ binds methylmalonic aciduria and homocystinuria type C protein (MMACHC), which decyanates or dealkylates vitamin B_12_ depending on its form [20,21]. MMACHC, with the assistance of methylmalonic aciduria and homocystinuria type D protein (MMADHC), carries MMACHC-refined cobalamin to methionine synthase or to mitochondria, with the decision of fate governed by MMADHC [22]. In mitochondria, methylmalonic aciduria type B protein (MMAB) converts cobalamin to AdoCbl in an ATP-dependent manner and delivers it to methylmalonyl-CoA mutase (MMUT) [23], while methylmalonic aciduria type A protein (MMAA) controls the correctness of cobalamin forms inserted into MMUT [24].

Free vitamin B_12_ and vitamin B_12_ that has not been absorbed in the small intestine is utilized by resident bacteria further along the digestive path. A significant part of the human gut microbial community (estimated at ~45–55%) relies on external vitamin B_12_, about 20–40% of human gut microbes are capable of its synthesis (although corrinoids may be needed as precursors), and some do not use it in homeostasis [25,26]. Bacterial B_12_-dependent enzymes catalyze isomerization, dehalogenation, deamination, dehydration, and methyl transfer reactions [10]. Cobalamin synthesized by bacteria is not a significant source for humans because most of the bacteria reside downstream of the digestive path with respect to host B_12_ receptors and synthesized quantities are small with respect to daily intake needs [3,26]. Vitamin B_12_ rather shapes the microbiota in the gut by attenuating the metabolism of affected bacteria [3,11].

Vitamin B_12_ has complex absorption machinery in the body, and during aging, some parts may deteriorate, thus decreasing B_12_ bioavailability (Figure 1). Atrophic gastritis, a form of chronic age-associated stomach inflammation, reduces gastric acid secretion necessary for vitamin B_12_ release from proteins [27]. Moreover, old patients have lower absorption in the small intestine and decreased renal re-absorption of vitamin B_12_ due to a change in expression of the receptor for the vitamin B_12_–IF complex in enterocytes and the kidneys [28]. The brains of old subjects have lower levels of MeCbl and AdoCbl than young subjects, despite normal levels of vitamin B_12_ in blood, thus indicating deterioration of vitamin B_12_ import into cells and processing [29].

## 3. B_12_-Dependent Proteins

Bacteria have a dozen B_12_-dependent proteins, while in mammals, only two proteins utilize vitamin B_12_ [3]. Methionine synthase (MS) produces a universal methyl donor, S-adenosyl-methionine (SAM), which is used in most methylation reactions. Mitochondrial methylmalonyl-CoA mutase (MMUT) converts methylmalonyl-CoA to succinyl-CoA in the Krebs cycle for the utilization of branched carbon chains derived from lipids and amino acids.

### 3.1. Methylmalonyl-CoA Mutase

**Figure 2:**
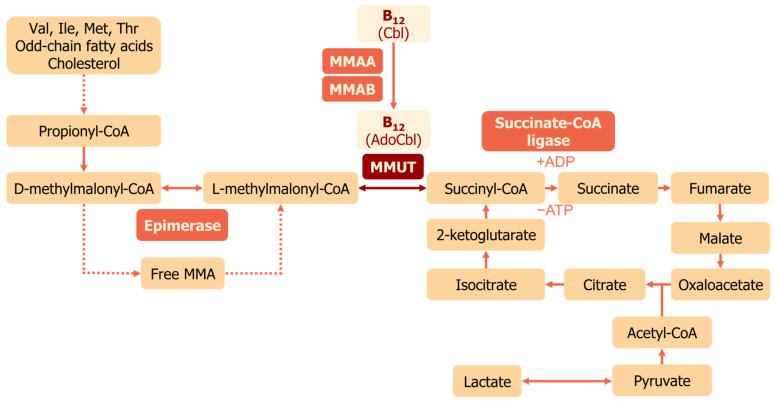
MMUT function in mitochondria. Propionate formed from the catabolism of branched amino acids and odd-chain fatty acids is converted to D-methylmalonyl-CoA. Methylmanolyl-CoA epimerase isomerizes D-methylmalonyl-CoA to the L-form. L-methylmalonyl-CoA is the substrate for MMUT, converting it to succinyl-CoA, which enters the TCA cycle. Abbreviations: MMA—methylmalonic acid, TCA cycle—the tricarboxylic acid cycle.

Methylmalonyl-CoA mutase is a mitochondrial enzyme that uses vitamin B_12_ in the form of AdoCbl as a cofactor [30,31]. MMUT converts L-methylmalonyl-CoA to succinyl-CoA [32], which then enters the tricarboxylic acid (TCA) cycle (Figure 2). Methylmalonyl-CoA is derived from the catabolism of branched-chain amino acids, lipids, and cholesterol side chains; thus, MMUT connects this metabolism to the TCA cycle (Figure 2). Human MMUT is a homodimer, and every subunit has N- and C-domains [33]. Each subunit of MMUT accepts one molecule of AdoCbl [30]. The N-domain binds the substrate, and the C-domain binds AdoCbl [33]. They are connected through a linker, and the active site is located at the N-/C-domain interface. MMAA ensures selective binding of AdoCbl and no other cobalamins to MMUT [33]. MMUT follows a radical generation mechanism: the Co-C bond in AdoCbl is cleaved along with the generation of a 5′-deoxy-5′-adenosyl radical for isomerization of methylmalonyl-CoA [34]. Reactivation of MMUT occurs due to MMAA, which exchanges an oxidized cofactor [24]. MMAA induces the release of the inactive cofactor and restores MMUT activity through their complex formation.

Disturbed functioning of MMUT due to scarcity of vitamin B_12_ leads to impaired mitochondrial function caused by methylmalonyl-CoA accumulation and subsequent aberrant acylations of proteins. It has been shown in model mice with liver-specific *MMUT* knockout and patients with methylmalonic aciduria, a disease caused by mutations in genes encoding vitamin B_12_-interacting proteins [2]. In this context, the activity of the mitochondrial enzyme carbamoyl phosphate synthetase-1 (CPS-1), which participates in the urea cycle, is severely compromised. The number of mitochondrial DNA copies and transcription of genes coding for the electron transport chain are reduced due to reduced activity of the associated transcription factor and the polymerase. However, the levels of these proteins (mitochondrial transcription factor A, TFAM, and DNA-directed RNA polymerase) do not change. The glycine cleavage system is compromised due to prevention of lipidation of glycine cleavage H protein (GCSH). All of these abnormalities are caused by an increase in the acylations of the corresponding enzymes caused by MMUT malfunction.

### 3.2. Methionine Synthase

**Figure 3:**
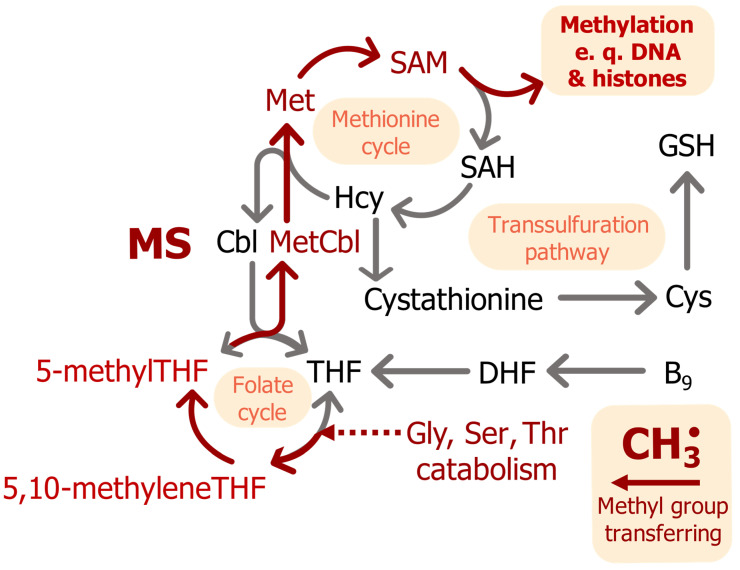
The MS catalytic cycle and its connection to cellular metabolism. Red arrows indicate the pathway of a methyl radical. Abbreviations: MS—methionine synthase, THF—[5,6,7,8]-tetrahydrofolate, DHF—[7,8]-dihydrofolate, Hcy—L-homocysteine, SAH—S-adenosyl-L-homocysteine, SAM—S-adenosyl-L-methionine, GSH—reduced glutathione, MetCbl—MeCbl, Cbl—cobalamin, CH_3_˙—methyl radical.

Methionine synthase (5-methyltetrahydrofolate-L-homocysteine S-methyltransferase, EC 2.1.1.13; MS) is a cytosolic enzyme that uses MeCbl as a cofactor [35]. The gene has ubiquitous expression, with the highest numbers of transcripts in epithelial tissues and the lowest in the brain [36]. Free vitamin B_12_ regulates MS expression through a feedback loop with a B_12_-dependent internal ribosome entry site (IRES) before a coding sequence for MS [37]. MS from *E. coli* is the most-studied MS; it has 53% homology to the human enzyme [38]. The protein consists of five domains: Cob—a cobalamin-binding domain, Hcy—an L-homocysteine-binding domain, Fol—a folate-binding domain, Act—an S-adenosyl-L-methionine-binding domain, and Cap—a cobalamin-binding domain. The Hcy, Fol, and Cap domains sequentially interact with Cob during the MS catalytic cycle [39,40]. The MS catalytic cycle consists of three steps [41,42,43,44]. In the first step, a cobalamin molecule, the MS cofactor, accepts a methyl group from 5-methyltetrahydrofolate and then transfers it to the sulfur atom in L-homocysteine to produce L-methionine, thus acting as a methyl donor. In the second step, the cobalt ion in the cobalamin is methylated with 5-methyltetrahydrofolate and covalently binds to the N_5_ atom of tetrahydrofolate. In the third step, tetrahydrofolate dissociation occurs by homolytic cleavage of the Co-N bond and the return of the cobalamin to its initial state. Oxygen and physiological oxidants may cleave the bond between the cobalt ion and the methyl group. In this form, cobalamin cannot enter the catalytic cycle, and the catalytic cycle is terminated after about 2000 repeats. A restoration of enzyme function requires reduction of MS by NADPH reductase (EC 1.16.1.8) and reactivation of cobalamin [45]. Produced L-methionine is a substrate for methionine adenosyltransferase, which converts it to S-adenosyl-L-methionine (SAM)—a universal methyl donor (Figure 3). Demethylation of 5-methyltetrahydrofolate in the folate cycle provides a methyl radical for cobalamin and increases the level of metabolically active B_9_ (folate) (Figure 3) [46,47]. The MS cycle is dependent on glycine, serine, and threonine catabolism as it supplies 5-methyltetrahydrofolate.

Genes of SAM-dependent enzymes are highly present in eukaryotic genomes; these enzymes occupy about 1% of total eukaryotic proteins, including DNA- and RNA-methyltransferases and histone methyltransferases [48,49]. Methylation of phospholipids is the main way in which SAM is consumed [50]. This process is used for the regulation of cellular methylation potential and creates methyl “sinks” [50]. Methylation reactions are competitively inhibited by their coenzyme product, S-adenosyl-L-homocysteine [51]. Phospholipid methylation also occurs after activation of dopamine receptor D4.4R in an SAM-dependent reaction involving a methionine in the receptors [52]. Thus, the activity of D4.4R also regulates other methylation processes, including DNA methylation [53].

When there is an excess of physiological oxidants (oxidative stress), methionine is misincorporated instead of other amino acids in protein synthesis to provide new functions for proteins or scavenge oxidants in the protein-bound form [54,55]. Free methionine may be oxidized and reduced with the methionine sulfoxide reductase A, which is capable of reducing methionine-(S)-sulfoxide in the free form [56].

A participant in the methionine cycle, homocysteine, is a precursor for glutathione in the transsulfuration pathway. Glutathione is a tripeptide crucial for cellular reduction–oxidation homeostasis [57]. Glutaredoxin enzymes use glutathione for the reduction of disulfide bonds in cells. In healthy cells, a high ratio of reduced glutathione (GSH) to oxidized glutathione (GSSG) (close to 98%) helps to maintain intracellular thiols in the reduced state [58].

## 4. Nucleic Acids and Histone Methylation

Vitamin B_12_ induces the production of S-adenosylmethyonine (SAM), which is a methyl donor in DNA and histone methylation (Figure 3). DNA methylation is the process of transferring a methyl group from the donor molecule (SAM) to nucleotide bases, which is catalyzed by SAM-dependent DNA-methyltransferases. In mammalian somatic cells, the methyl group is usually covalently bound to C_5_ of cytosine in CpG dinucleotides, where about 70–80% of CpG dinucleotides in somatic cells are methylated [59]. 5-Methylcytosine can populate ~1% of cellular DNA [60], and some anticancer drugs, such as cisplatin, can specifically interact with it [61]. Cytosine methylation in CpA and CpT dinucleotides was found in stem cells [62]. 6-Methyladenosine is another type of DNA methylation in mammalian cells [63]. It is detected in excess amounts in tumor cells of the esophagus and liver [64,65]. Thus, various nucleotides in DNA can be methylated.

DNA methylation plays a regulatory role in gene expression. The main mechanisms of gene repression due to methylation are inhibition of the binding of transcription factors to DNA in hypermethylated regions and the binding of such regions to repressor methyl-binding proteins [66]. The strongest repression of gene expression occurs when its promoter or the first exon are methylated [67]. Methylation of a gene body positively correlates with its expression level in dividing cells [68]. DNA demethylation has opposite effects on gene expression. Global DNA demethylation mainly occurs during early embryogenesis, stem cell differentiation, and reprogramming [69]; however, aging is accompanied by DNA demethylation [70]. DNA is demethylated with Fe^2+^/α-ketoglutarate-dependent dioxygenases [71]. α-Hydroxyglutarate is an inhibitor of these enzymes. Mutations in the *IDH1* or *IDH2* genes lead to the reduction of α-ketoglutarate to α-hydroxyglutarate and subsequent DNA hypermethylation, including tumor suppressor genes, and oncological transformation of cells [72]. ALKBH1 demethylates 6-methyladenosine in DNA and RNA [63,73].

Defects in DNA methylation in cells lead to impaired gene repression and aberrant expression, which disrupt their homeostasis, increase tumor occurrence, and promote cellular aging. For example, methylation of the transposable repetitive sequences Alu and LINE-1 is reduced with age, similarly to cancer cells [70,74,75]. In tumor cells, unlike in normal cells, the methylation regulation by long non-coding RNA is impaired [76]. Methylation of microRNA regulatory regions strictly controls their expression in normal cells, while at the same time, tumor cells [77,78] and aging cells [79] are characterized by abnormal expression of microRNAs associated with relevant processes. Moreover, aging is associated with a drastic change in DNA methylation patterns [80]. Thus, the balance between DNA methylation and demethylation plays a critical role in the normal homeostasis of the cell.

Dynamic post-translational modifications of histones provide regulation of chromatin structure and gene expression [81]. Histones are highly conserved proteins that organize eukaryotic DNA into nucleosomes. Currently, more than 25 different types of post-translational modifications of histones, which can affect their various amino acid residues, are known.

Methyl groups on histones are specific marks for enzymes that interact with DNA and change the structure of the chromatin. In humans, histone methylation is catalyzed by dozens of SAM-dependent methylases [82]. The regulatory function of methylation depends on the position at which it occurs (Table 1). Some modifications may play an ambiguous role, for example, H3K79me3 [83,84], and there is an interplay and correlation between histone post-translational modifications [85]. SAM deficiency may cause impaired histone methylation and loss of heterochromatin, including age-associated loss [86]. Positions H3K4, H3K36, and H3K79 are most frequently methylated, and inhibition of these processes leads to a 2–5-fold increase in intracellular SAM levels [50], which indicates a significant need of SAM for histone methyltransferases. The modification H3K36me3 is a hallmark for exons [87], where it prevents transcription initiation outside promoters [88]. Methylation of DNA regions is specifically inhibited in the presence of H3K4 methylation [89]. For the process of gene imprinting in mammals, the efficiency of the methylation processes of both DNA and histones is important. A knockout of the *DNMT3L* (DNA (cytosine-5)-methyltransferase 3-like) gene leads to a loss of the allele specificity of the histone repressive modifications H3K9me3, H4K20me3, and H2A/H4R3me2 in imprinting control regions [90].

Histone acetylation is associated with a transformation of heterochromatin regions into euchromatin and an increase in their transcriptional activity. Deacetylation of histones bound to a DNA region is mechanistically associated with hypermethylation of the CG-rich stretch of this region: the methyl-CG-binding protein meCP2 binds to hypermethylated DNA and recruits to nearby nucleosomes histone deacetylases and methyltransferases that methylate histones at the H3K9 position [95]. Histone deacetylation in humans is catalyzed by 18 different deacetylases, including 7 NAD^+^-dependent deacylases—sirtuins [96]. The strongest deacetylase activity is observed for sirtuins 1–3 [97]. In addition to removal of the acetyl group, sirtuins catalyze the removal of several other non-acetyl acyl modifications from histones and other proteins. SIRT5 has desuccinylase, demalonylase, demethylmalonylase, and other activities [2,98,99]. Sirtuins are involved in the processes of suppressing cellular aging by delaying replicative shortening of telomeres, maintaining genome integrity, and promoting DNA damage repair [100].

Manipulation of histone methylation and acetylation through deletion, inhibition, or overexpression of histone-modifying enzymes prolongs the lifespan of worms and flies and shows phenotypes of healthy aging in human cellular models (reviewed in [101]). DNA methylation and histone modifications, which are partially dependent on vitamin B_12_ availability, are involved in aging [102].

## 5. Consequences of Vitamin B_12_ Deficiency

**Figure 4:**
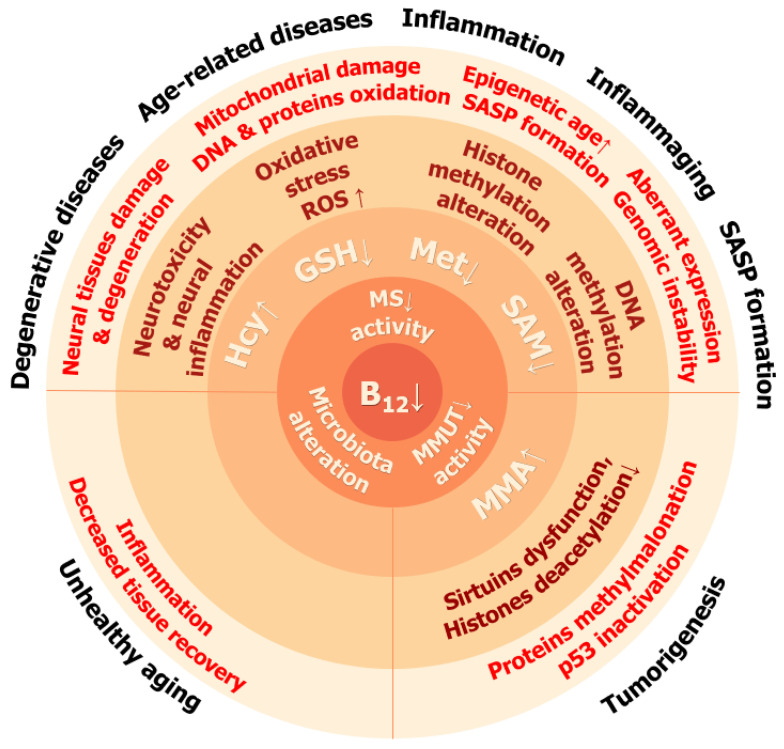
Consequences of vitamin B_12_ deficiency at cellular, tissue, and organismal levels. Arrows indicate direction of change: ↑—increase, ↓—decrease.

Vitamin B_12_ deficiency causes several consequences on organismal, cellular, and molecular levels which resemble some of the phenotypic consequences of aging and senescence (Figure 4).

Currently, there is no universal method for determining B_12_ deficiency for widespread application which could be considered the “gold standard” [103]. In most cases, total vitamin B_12_ levels are measured in serum. In clinical practice, measurement of total serum B_12_ is made using a chemiluminescence (ECL) assay by measuring the competitive binding of purified haptocorrin to vitamin B_12_ after its release from endogenous proteins [104]. This technique is available for widespread application due to its precision (detection is possible in the range of 50–2000 pg/µL), speed, and efficacy and the availability of high-tech automated systems [105]. A deficiency in biologically available B_12_ can be indirectly determined by the concentration of the protein that transports it, holotranscobalamin (holoTC) [103]. Concentrations of several small molecules increase due to a deficiency in active forms of B_12_: MMA (2-methylmalonic acid), due to insufficiency of the MMUT function, and Hcy (homocysteine), due to insufficiency of the MS function (Table 2), which may be quickly and accurately measured with automated systems [103]. However, the serum vitamin B_12_ concentration, whether detected directly or indirectly, may not correspond to the intracellular vitamin B_12_ concentration. Even with “adequate levels” of vitamin B_12_ in blood, concentrations of B_12_ forms inside cells can be significantly decreased [29]. In laboratory settings, HPLC-UV with internal standards [106] or HPLC strengthened with mass spectrometry or tandem mass spectrometry (HPLC-MS/MS), which do not require internal standards, are routinely used for measurement of vitamin B_12_. The advantages of HPLC are its high speed, accuracy, sensitivity, and specificity for various cobalamins, allowing quantitative measurements of cyanocobalamin, MeCbl, hydroxocobalamin, other cobalamins, cobinamides, and corrinoids. HPLC methods usually do not require difficult sample preparation [106]. Typically, HPLC and HPLC-MS have a two- to three-order lower limit of detection, thus excluding single-cell analysis for research purposes.

While in North America the fraction of the population with low serum B_12_ levels is not greater than 10%, in other regions such fractions can exceed 40% [107]. Marginal vitamin B_12_ deficiency is reported in 30–80% of the population (Table 2) [107]. In elderly populations around the world, rates of severe and marginal vitamin B_12_ deficiency are higher [107].

Vitamin B_12_ deficiency during gestation leads to a number of metabolic disorders in the offspring, which may reduce their lifespan. In Wistar rats, folic acid supplementation along with vitamin B_12_ deficiency during gestation reduces global DNA methylation and plasma and placental levels of ω-3 docosahexaenoic acid [108]. The offspring of rats fed a vitamin B_12_-deficient diet have a higher risk of developing obesity, insulin resistance, and elevated levels of triacylglycerides and glucose, which increases the probability of progression of metabolic and cardiovascular diseases with age [109]. They also have reduced resistance to oxidative stress and a lower birth weight, elevated levels of TNFα are found in the blood and adipose tissue, and there are increased levels of cortisol, leptin, and pro-inflammatory IL-6 in blood, while levels of adiponectin and IL-1β, on the contrary, are decreased. In offspring, changes in glucose metabolism and antioxidant status can be reversed with a return of serum B_12_ to physiological levels after fertilization. In the postnatal period, these changes can be partially, but not absolutely, reversed [109]. These phenomena may be based on a decrease in CpG methylation in non-coding regions before the onset of genes associated with the transport and metabolism of fatty acids, including mitochondrial genes, in the offspring of dams [110]. These consequences can be phenotypically and molecularly reversed when B_12_ levels in gestational females return to normal [109]. Similar effects have been observed in sheep. Offspring of sheep fed a vitamin B_12_-deficient diet and folate before fertilization are more likely to be overweight, antigen-hypersensitive, insulin-resistant, and to have high blood pressure than offspring of sheep fed a normal diet [111]. At the stage of fetal development, an altered methylation status of 4% of 1400 CpG islands is observed in sheep liver tissues, which may indicate an epigenetic mechanism for the offspring to acquire metabolic disorders due to B_12_ deficiency in the mother. Thus, vitamin B_12_ concentrations are very important in the early stages of ontogenesis.

Deficiency in MeCbl and AdoCbl in cells due to insufficiency of vitamin B_12_ supply to cells causes promiscuous non-enzymatic post-translational modifications. Scarcity of MeCbl and consequent deterioration in methionine synthase function increase amounts of homocysteine. Homocysteine may be converted to homocysteine thiolactone, which non-enzymatically modifies ε-amino groups of lysines [112], including those on histones H3K27, H3K36, and H3K79 responsible for chromatin structure [113]. In the brains of mice with decreased B_12_ in their food, expression of histone-modifying enzymes (e.g., H4K20-methylating and Zn^2+^-dependent histone deacetylase HDAC4) increases [114], and the amount of oxidative stress markers (malondialdehyde and protein carbonyls) rises. Decreased activities of superoxide dismutase and the H_2_O_2_-degrading enzyme catalase in such brains lead to these oxidative post-translational modifications [114]. Moreover, vitamin B_12_ is capable of scavenging superoxide radicals with a rate similar to that of superoxide dismutase [115], and supplementation with B_12_ helps to reduce the consequences of oxidative stress [116]. A deficiency in AdoCbl, which is obligatory for the activity of MMUT, provokes accumulation of methylmalonyl-CoA. Deficiency in the MMUT, MMAA, and MMAB functions leads to a range of metabolic diseases named methylmalonic acidurias, which have an increased concentration of methylmalonic acid (MMA) in blood as a consequence [117]. MMA and its derivative organic acids cause damage to the central nervous system [118]. On a molecular level, in model mice with a liver-specific knockout of MMUT, abnormal methylmalonylation and malonylation are present on a few proteins [2]. Despite these proteins being mostly localized to mitochondria, some of them are present in other compartments, such as the plasma membrane, the nucleus, lysosomes, and others, indicating that such modifications are not restricted to the mitochondria. Deacylases capable of demethylmalonylaton and demalonylation, such as SIRT1 and SIRT5 [2,98], which have strong activity against methylamolnylation and propionylation, are acyl-modified due to the loss of MMUT activity, thus decreasing their function [2]. In liver samples of patients with MMA abundancy, the activity of several sirtuins, including SIRT1 and SIRT5, is substantially reduced [2]. SIRT5 has a preference for extended acyl groups, such as malonyl, succinyl, propyonyl, and methylmalonyl groups [2,98]; thus, it is not solely methylmalonyl modifications that may accumulate.

At the organismal level, the effects of severe chronic B_12_ deficiency primarily affect the nervous system, resulting in neurological disorders. Chronic vitamin B_12_ deficiency leads to behavioral abnormalities in female mice: with a moderate to severe vitamin B_12_ shortage in the diet, they exhibit more anxious behavior and less maternal care for their offspring than controls with a sufficient B_12_ level [114]. Abnormal maternal behavior in model mice and a reduction in postnatal period duration are observed when there is a decrease in the expression of the *MEST* gene due to disturbances in its imprinting, a process dependent on DNA methylation [119]. Impaired histone methylation due to serum B_12_ deficiency, which was assessed by measuring serum homocysteine and holotranscobalamin levels, led to a disruption of gene imprinting, in particular, to a decrease in the expression of the *MEST* gene in the placenta of mice [120]. In infectious diseases of the nervous system, such as meningitis, a therapy including vitamin B_12_ promotes neuronal survival and reduces the inflammatory markers CCR2, CCL3, and IL-1β: bacterial meningitis model rats maintained on a vitamin B_12_-supplemented diet had fewer apoptotic cells in the hippocampal dentate gyrus than those who did not receive it [121]. In treatment of viral infections in humans, additional amounts of B_12_ reduce the intensity of pain and subsequently reduce the risk of memory loss and concentration problems [122]. Neuronal damage caused by B_12_ deficiency may involve increased levels of homocysteine due to lower activity of MS, as in autistic subjects [29]. Accumulation of Hcy in cells increases the probability of their apoptosis and necrosis, which can be attenuated with NMDA and mGluR1 antagonists [123]. The risk of development of mental disorders, such as autism and schizophrenia, is increased if patients have mutations in the genes associated with vitamin B_12_ transport and one-carbon metabolism: methionine synthase (*MTR*), methionine synthase reductase (*MTRR*), transcobalamin (*TCN2*), and 5,10-methylenetetrahydrofolate reductase (*MTHFR*), reducing their activity [29]. There is an association between low concentrations of active forms of B_12_ in the brain and mental disorders such as autism and schizophrenia [29]. Indeed, even normal serum vitamin B_12_ levels do not guarantee the same cellular B_12_ concentrations. In older people, the total concentration of B_12_ isoforms in the brain decreases with age [29]. Moreover, the amount of MeCbl and AdoCbl decreases significantly, and the amount of OHCbl increases, regardless of the normal serum concentration of B_12_, which may indicate accumulating defects in vitamin B_12_ processing inside cells. In the brains of old subjects, levels of homocysteine were higher compared to young subjects, and methionine and SAM levels were lower [29]. There is a connection between decreased vitamin B_12_ levels and decreased cognitive function, as well as worsening symptoms of neurological and psychiatric diseases [124]. In the nervous system, DNA methylation is associated with memory. The age-related decline in the ability to remember information and learn correlates with a decrease in global neuronal DNA methylation [125], and overexpression of Dnmt3a DNA methylase restores cognitive function in aged mice [126]. Since methylation may not be efficient enough when the concentration of SAM in neurons decreases [127] and a decrease in SAM levels is observed with premature aging and numerous age-associated diseases [128], these phenomena may be related to each other and to a deficiency in B_12_, a cofactor of methionine synthase (Figure 3). Vitamin B_12_ deficiency worsens the prognosis for patients with age-associated neurodegenerative diseases. Patients with Alzheimer’s disease were found to have higher serum concentrations of Hcy and lower levels of folate and B_12_ than the healthy control group [129]. Reduced serum levels of vitamin B_12_ are also associated with the rapid progression of Parkinson’s disease, worsening cognitive ability to move, and cognitive impairment [130]. On the other hand, older patients with these conditions often have impaired B_12_ absorption, so whether B_12_ deficiency is a cause or consequence of age-related frailty is not yet established [124].

An increase in the level of B_12_ in blood plasma may indicate pathological processes in the organism. Solid tumors, myeloproliferative diseases, tumor metastases in the liver, and liver and kidney diseases are possible consequences of elevated serum B_12_ levels [131]. In particular, long-term elevated plasma B_12_ concentrations are significantly associated with the occurrence of solid tumors [4]. On the other hand, higher levels of Cbl in plasma are associated with a higher antioxidant capacity, which may be due to direct radical scavenging capacity and also due to participation in the transsulfuration pathway for glutathionine synthesis [132], which is reduced in elderly people [29].

Vitamin B_12_ is essential for cellular homeostasis, and its deficiency causes many abnormalities, such as oxidative stress, impaired DNA and histone modifications, neurological disorders, and disturbance of signaling pathways such as sirtuin pathways.

## 6. Role of B_12_ in Aging, Senescence, and Inflammation

Aging leads to a gradual decline in the organism’s performance, which is reflected on cellular and molecular levels. Prominent hallmarks of aging include pronounced epigenetic changes, impaired mitochondrial function, cellular senescence, chronic inflammation, and others [133]. It is known that deficiency in or low levels of vitamin B_12_ correlate with worsening symptoms of age-related conditions, such as sarcopenia and dynapenia, cognitive decline, frailty syndrome, and cardiovascular diseases (reviewed in [6,124]). Global expression of the vitamin B_12_ transporting and processing proteins MMAA, MMUT, MMADHC, and haptocorrin decreases during aging [134], which potentially impairs the absorption of vitamin B_12_ from the intestine, reduces its processing on the way to MS and MMUT, and additionally decreases the activity of MMUT. Below, we discuss aging- and senescence-associated mechanisms caused by (sub-)deficiency in vitamin B_12_. However, elevated levels of plasma vitamin B_12_ increase the overall risk of mortality in elderly individuals [135,136,137], indicating that there is an optimal range of vitamin B_12_ concentrations_._

### 6.1. Vitamin B_12_ and Its Role in the Formation of the Senescence Phenotype

Cellular aging is a biological process that leads to a gradual increase in the probability of cell death due to a progressive loss of normal cell function. It consists of two stages: replicative aging, which causes a cell to lose its ability to divide, and senescence, a program induced by cell cycle arrest characterized by a pro-inflammatory phenotype and prevention of proliferation [138]. Telomere shortening and end-to-end chromosome joining in aged cells are the hallmarks of replicative aging [139]. Senescence is characterized by changes in DNA methylation patterns and histone post-translational modifications, aberrant gene expression due to large-scale chromatin rearrangement, disturbances in signaling cascades, mitochondrial dysfunction and related oxidative stress, mitochondrial damage, and decreased NAD+ levels [140]. Senescence is an adaptive mechanism rather than a cause of aging [141].

Senescent cells have features that are not typical of “young” ones. Senescent cells are characterized by irreversible arrest of the mitotic cycle and dysregulation of signaling through multiple pathways (sirtuin, IGF-1, mTOR, AMPK, FOXO, and NF-kB pathways). Cells that have entered the state of senescence are characterized by specific and increased activity of lysosomal β-galactosidase (senescence-associated β-galactosidase, SA-β-gal) at pH = 6, and this is used for their quantitative and qualitative determination in laboratory experiments [142,143]. Protein p16 is a product of the tumor suppressor gene *CDKN2A*, an inhibitor of cyclin-dependent kinases 2A and 4, blocking the transition of cells from the G1 to S phases [144]. The expression of the tumor suppressor gene significantly increases with age in most mammalian tissues, and the level of expression in T lymphocytes is a biomarker of aging [145]. Hypermethylation of the promoter of the gene encoding p16 protein increases cell resistance to oxidative stress and prevents premature cell aging induced by oxidative stress, as shown in the example of keratinocytes [146]. The p21 protein is an inhibitor of cyclin-dependent kinases 1 and 2, and its expression results in the arrest of the cell cycle at the G1 or G2/M phases [147]. The senescence state can be observed even without p16 expression if the p21 expression level is high, which has been shown in myocytes [148] and fibroblasts [149]. The protein p53 arrests the cell cycle in G1 and induces p21 expression as a transcription factor [150]. Senescent cells reduce p53 activity [151]. Senescent cells with low p16 expression levels proliferate after p53 inactivation, whereas senescent cells with high levels of p16 expression do not, even when p53 is inactivated [152].

One of the most important characteristics of senescent cells is the change in the secretory phenotype to the senescence-associated secretory phenotype (SASP), which includes increased secretion levels of interleukins (ILs), chemokines, growth and regulatory factors, and metalloproteinases [101,153,154,155]. SASP affects surrounding cells in a variety of ways. For example, some factors may act by inducing cellular senescence, thus limiting tumor progression. SASP promotes the elimination of senescent cells by stimulating the immune system and promoting tissue repair. In that respect, SASP is considered to be a beneficial response of senescent cells in organisms [156,157]. On the other hand, SASP has negative consequences for the organism under normal and pathological conditions. Some SASP factors, depending on the cellular context and environment, have deleterious effects on organisms, such as tissue inflammation and tumor progression [158,159]. In tumors, SASP has been shown to influence various processes: the initiation of epithelial–mesenchymal transition [160,161], induction of stemness [162,163], local tissue invasion [160,161,164], angiogenesis [165], fibroblast activation [166], immunosuppression [159,167], enhanced metastasis [168], and resistance to therapy [169,170,171,172].

Appropriate regulation of cellular senescence and SASP will be beneficial for human health because the elimination of senescent cells reduces age-related disorders and extends the lifespan of organisms [173]. To prevent senescent cell accumulation in tissues, the removal of senescent cells from these tissues may be achieved with the use of senolytics—molecules which selectively eliminate senescent cells [174]. However, the complete removal of senescent cells in tissues causes impaired regeneration of lung and liver tissues, although it improves bone regeneration [175].

Another approach for reducing the number of senescent cells in tissues is to reverse their transition to a senescent state by inducing their pluripotency using Yamanaka factors (OSKM: Oct3/4, Sox2, c-Myc, and Klf4) and subsequent redifferentiation [176]. Induced pluripotent stem cells (iPSCs) are morphologically indistinguishable from human embryonic stem cells (hESCs) and can be reprogrammed from senescent cells. Such cells have comparable telomere lengths and can be re-differentiated into rejuvenated tissue-specific cells [177]. A high level of p16 expression in senescent cells is a factor limiting the efficiency of reprogramming, and the removal of such cells from the tissue significantly increases reprogramming efficiency [178]. Additional supplementation with vitamin B_12_ improves reprogramming in model mice with tetracycline-inducible expression of OSKM [179].

Maintaining the state of senescence requires adaptation, which is achieved by deep epigenetic rearrangement. These changes are necessary, on the one hand, to maintain permanent cell cycle arrest and avoid apoptosis and, on the other hand, to implement the SASP. In this context, senescent cells are characterized by the formation of specific regions of heterochromatin called senescent-associated heterochromatin foci (SAHF) [180]. However, SAHF are not typical for all senescent cells [102]. SAHF are defined as DAPI-dense nuclear regions characterized by the presence of a central core of condensed chromatin enriched in H3K9me3 and macroH2A. The core is bordered by a peripheral ring containing H3K27me3 [181]. SAHF formation requires the presence of p16 and represents a deep and targeted reorganization of heterochromatin [182]. SAHF formation promotes both gene activation and gene repression. There are other epigenetic features, such as senescence-associated satellite stretching, reactivation of mobile elements and endogenous retroviruses, and lamin modifications, which are reviewed elsewhere [74].

During senescence, the epigenome undergoes significant and consistent changes that are necessary for cellular adaptation. DNA and histone methylation are important changes that together provide dynamic epigenetic control over gene expression [183]. To implement these changes, cells utilize the machinery to add methylation marks to DNA and histones. To highlight the link between B_12_ and global epigenetic rearrangements in senescent cells, we will discuss the specifics of this program in senescent cells.

Impaired histone modifications appear to play a special role in senescence and aging. Accumulation of H3K9me3/H3K27me3/macroH2A during SAHF block formation is typical for senescent cells undergoing oncogene-induced senescence [184]. Meanwhile, a global decrease in H3K9me2/3 and H4K20me levels but an increase in H3K9me1 levels in gene bodies characterizes replicative senescence in human fibroblasts [185]. In rat hepatocytes, trimethylation of histone H4 at position K20 increases with age [186]. The prevalence of the epigenetic modification H4K20me3 near the promoters of genes is associated with the SASP in cancer cells and reversibly terminates the mitotic cycle [187]. The rarity of H4K20me3 in the same positions in senescent cells may indicate an epigenetic mechanism for the transition of cells to this state when the H4K20 methylation process is disrupted, which may be a consequence of disturbed one-carbon metabolism (Figure 1) [187]. A decrease in H3K9 methylation is observed in Hutchinson–Gilford progeria (premature aging), and the depth of this decrease is proportional to an increase in the level of a defective nuclear protein, lamin A [188,189,190].

The onset of replicative senescence is linked to telomere shortening. Telomere shortening is often a by-product of either oxidative stress or inflammation. Micronutrients with antioxidant properties can protect telomeres from such damage. Vitamin B_12_ is essential for maintaining DNA integrity through nucleotide biosynthesis and methylation. Plasma B_12_ levels are associated with telomere length [191,192]. Vitamin B_12_ supplementation in women was associated with an increase in telomere length [193]. In addition, both low B_12_ levels associated with Hcy accumulation and high B_12_ levels associated with inflammation may influence telomere shortening [191]. These experimental data also reveal an unrecognized role of B_12_ in prevention of telomere shortening and regulation of senescence-associated processes through a positive feedback loop: since B_12_ is required for DNA methylation and telomere length is epigenetically regulated by DNA methylation, B_12_ levels appear to be directly reflected in telomere length [194].

A deficiency in vitamin B_12_ leads to the appearance of cellular senescence. Vitamin B_12_-deficient astrocytes have increased levels of SA-β-gal. Furthermore, overexpression of p16 and p21 in this model was conditioned by B_12_ deficiency [195]. Disruption of DNA synthesis due to cobalamin deficiency can probably induce p21 expression [196]. hESCs grown in a medium deficient in vitamins B_6_, B_9_, and B_12_ exhibit a number of phenotypic changes: cell disintegration, expression of senescence-associated β-galactosidase, reduced metabolic activity, and smaller size and number. These changes can be partially reversed by increasing the level of vitamins B_6_, B_9_, and B_12_ from 10–30% of their physiological concentrations to 70%; however, only hESCs that are moderately damaged by vitamin deficiencies are subject to such restoration [197]. Telomere length and mitochondrial DNA copy number decrease as cells age in inverse proportion to their B_12_ levels [198].

When MMUT function is deteriorated, e.g., in methylmalonic aciduria or in vitamin B_12_ deficiency, non-enzymatic post-translational modification of proteins such as malonylation and methylmalonylation may change the functions of a few proteins [2]. Such proteins are involved in the innate immunity response, maintenance of nuclear and mitochondrial DNA, RNA stability, and cell division control [2]—the features that change during aging [101]. Some of them directly interact with senescence-associated proteins, for example, those involved in the p53 signaling pathway, yet others, such as MMP13, are part of the SASP. Sirtuin SIRT5 removes acyl post-translational modifications, including those on histones [98]. However, in cases of deterioration of MMUT function, its activity is reduced due to MMUT modification [98]. Levels of global lysine malonylation are higher in the brains of older mice than in those of young mice [199], including proteins with molecular masses close to those of histones. Since histone malonation, especially in H2BK5, is regulated by SIRT5 in the mouse brain and liver, SIRT5 may have a significant role in aging [199]. Vitamin B_12_ deficiency also causes ER stress due to impaired SIRT1 deacetylation of heat shock factor 1 (HSF1) [200], which can be reversed by the drug metformin [201]. An increase in lifespan, which occurs in model yeast with calorie restriction, does not occur in cells with a mutated *SIRT2* homolog gene [202]. During aging, MMA accumulation in blood (which is a characteristic of impaired MMUT function) promotes transformation of cells into aggressive tumor-like states [203]. Thus, decline in MMUT function due to scarcity of AdoCbl leads to impaired mitochondrial function, which is a feature of senescence.

It is important to note that SASP expression is also directly regulated by epigenetic rearrangements during SAHF formation. At the same time, as we have pointed out, it is SASP that is the major negative component of the presence of senescent cells in tumors. It is possible that the effects on vitamin B_12_ and the understanding of its fundamental effects on SAHF formation will help to regulate epigenetic rearrangements during senescence in the future. This will help to avoid the negative effects of senescent cells in cancer treatment. It is currently known that any type of cancer therapy (immunotherapy, chemotherapy, or radiotherapy) leads to an increase in the pool of senescent cells.

### 6.2. Vitamin B_12_ and Epigenetic Age

DNA methylation is used for the determination of the “biological” age of mammalian cells because methylation patterns change with age [204]. Thus, it can be assessed by “epigenetic clocks” by assessing the levels of hypo- and hypermethylation of certain CG regions of the genome [80,204].

In vitro and in vivo, loss of epigenetic information underlies the transition to the senescent state of mammalian cells, which is confirmed by an increase in the levels of its epigenetic marks during aging induced by DNA double-strand breaks (DSBs), such as hypomethylation of H3K27 and its hyperacetylation, and increase in “epigenetic age” [205]. Some of this information is lost due to DNA double-strand break repair mechanisms, even if mutations are not introduced in DNA. Induction of aging by double-strand DNA breaks in mouse cells doubled their epigenetic age with respect to control mice, and after the introduction of OSK factors, the epigenetic age of the mice was reduced by 57%. At the phenotypic level, mice subjected to such induced aging exhibit typical aging hallmarks. In mouse fibroblasts subjected to DSB-induced senescence, histone modification of H3K27me3 decreases, while that of H3K27ac increases. The described changes can be reversed with a time-limited induction of their pluripotency during epigenetic reprogramming with OSK factors [205]. Additional B_12_ amounts improve the efficiency of cellular reprogramming using OSKM factors in OSKM mice and promote faster regeneration of the intestinal epithelium after ulcerative colitis induced by sodium dextran sulfate [179]. The limiting factor for successful cellular reprogramming is vitamin B_12_, deficiency in which leads to SAM deficiency, which can impede the global rearrangement of DNA and histone methylation patterns, including H3K36me3 histone modification [51,179]. H3K36me3 prevents cryptic transcription, which is one of the hallmarks of senescent cells; thus, additional vitamin B_12_ increases translation fidelity during reprogramming [88,179,206].

Whether dietary supplementation with vitamin B_12_ increases or decreases epigenetic age is controversial. It has been known for more than three decades that global DNA methylation levels decrease with age [207,208,209] as well as in the case of impaired transport of vitamin B_12_ into cells [210]. A meta-analysis shows that supplementation of B_12_ with folic acid in adults increases global DNA methylation and changes gene-specific methylation status [211]. In a randomized, double-blinded, controlled clinical trial, after 1 year of treatment, older patients who received supplementation with calcium and vitamins D, B_6_, B_9_ and B_12_ versus calcium and vitamin D tended to have a higher epigenetic age based on the “epigenetic clocks” developed by Weidner and colleagues [212,213]. However, the effect was more pronounced for initially epigenetically “younger” individuals whose epigenetic age was lower than the chronological age at the baseline. This may indicate an influence on DNA methylation and not necessarily on the aging process, since potential confounders, such as 5,10-methenyltetrahydrofolate reductase polymorphism (*MTHFR* C677T), which causes increased homocysteine and decreased folate concentrations, were not taken into account. In another study with elderly volunteers, supplementation with folic acid and vitamin B_12_ increased overall DNA methylation, and changes in epigenetic age were dependent on *MTHFR* genotype and sex [214]. Women with *MTHFR* C677T exhibited the most pronounced reduction in expected and observed epigenetic age [214]. Despite the study design benefiting from the exclusion of individual-specific confounders due to the collection of blood samples before and after the intervention, the study lacked a placebo group. In another study, supplementation with vitamin B_12_ and folic acid for 2 years revealed six significantly differentially methylated regions in elderly individuals in comparison to the placebo group [1]. In the study, participants with either *MTHFR* genotype were equally distributed between the groups. Moreover, the methylation status of 425 regions correlated with the serum vitamin B_12_ level [1]. Long-term intake of folic acid and vitamin B_12_ in elderly people leads to changes in DNA methylation of genes involved in normal development and tumorigenesis [1]. Interestingly, in the data provided by Kok et. al., methylation of the 1500 bp upstream region of the gene *PRKDC*, a DNA damage sensor, is dependent on the serum holotranscobalamin level, and the product of *PRKDC* is methylmalonyated in the mouse model of MMUT deficiency [2]. In worms, feeding with MeCbl or AdoCbl decreases reproductive age [215]. However, in infants, higher B_12_ levels and lower levels of Hcy in plasma are correlated with lower epigenetic age [216].

Thus, adequate serum B_12_ levels fit the antagonistic pleiotropy theory of aging, a beneficial metabolite at a young age becoming detrimental at an older age.

### 6.3. Role of B_12_ in Cytokine Regulation and Inflammation

There is growing evidence that B_12_ levels are associated with markers of inflammation. Patients with high B_12_ levels and long telomeres have significantly lower levels of inflammatory markers such as IL-6. The authors of a study proposed that patients with high B_12_ levels and systemic inflammation have an increased risk of death due to accelerated telomere shortening [191]. In patients and in naturally aged mice, a decreased level of serum vitamin B_12_ is correlated with increased inflammatory markers, such as proinflammatory cytokine IL-6 and reactive C protein [5]. Increased concentrations of IL-6 are associated with all-case mortality in elderly individuals [217]. We hypothesize that this may be related to the senescence and production of SASP. IL-6 is a major component of SASP and a cytokine that plays a negative role in oncogenesis [218,219]. However, data from human cohorts are contradictory. While some studies have found an association, others have not. Lee et al. reported a reverse association between low vitamin B_12_ status and high IL-6 levels among people with diabetes [220]. In contrast, treatment of 150 women with vitamin supplements containing vitamin B_12_ for an average of 7 years had no effect on IL-6 and markers of endothelial dysfunction [220]. In the literature, vitamin B_12_ is discussed as an anti-inflammatory agent because of suppression of nuclear factor-κB (NF-kB), inhibition of nitric oxide synthase, and stimulation of oxidative phosphorylation [221,222]. The major cytokine that triggers the NF-kB pathway is TNF [223]. Increased blood levels of cobalamin are associated with inflammatory diseases and TNF levels [224]. Cobalamin levels also modulate TNF levels in the cerebrospinal fluid of patients [225]. This is complemented by several studies in cobalamin-deficient rats showing increased levels of some neurotoxic molecules, including TNF, in cerebrospinal fluid [226,227]. TNF plays an important role in inflammatory reactions and aging processes and in the activation of the SASP program [228,229].

Vitamin B_12_ may also alleviate inflammation in elderly individuals in another way. Itaconate is converted from aconitate derived from the Krebs cycle by the enzyme IRG1 (Aconitate decarboxylase 1, ACOD1) mainly expressed in macrophages [230]. Itaconate in its acid form is cell-permeable since the intracellular concentration of itanocyl-CoA, a product of itaconate, increases after incubation of cells with media supplemented with itaconate [231]. Itanocyl-CoA and citramyl-CoA bind to the active site of MMUT instead of methylmalonyl-CoA and irreversibly damage MMUT-bound AdoCbl [231]. This also prevents the damaged enzyme from being repaired [232]. Inability to recover MMUT and AdoCbl will deplete cobalamin inside the cells and disturb their metabolism. Given that itaconate may pass through the cell membrane and travel to adjacent cells, it will cause a local intracellular reduction in AdoCbl if the affected cells express IRG1. There is chronic, low-grade inflammation in aging, which is known as inflammaging [233]. In macrophages of aged mice, expression of Irg1 is higher than in young mice and leads to accumulation of itaconate inside cells [234]. Dietary supplementation of vitamin B_12_ together with other essential micronutrients decreases inflammaging [235]. Inflammaging may aggravate or even partially cause the consequences of cellular vitamin B_12_ deficiency.

Taken together, these data suggest that cobalamin modulates the expression of certain cytokines and growth factors, but few studies have been conducted at the cellular level as opposed to in human groups. This is due to the availability and widespread use of vitamin B_12_ as a nutritional supplement.

### 6.4. Microbiota, Vitamin B_12_, and Aging

Since vitamin B_12_ is ingested from food in the intestine, microbial community members compete for vitamin B_12_ with the host and each other (Figure 1). Only few species in human microbiota are able to produce cobalamin themselves from preceding molecules, namely, cobanamides [9]. During aging, microbial communities as well as metabolic pathway activities alter [236]. The composition of the microbiota has been used for age prediction and correlates with diet [237]. A more recent model for age prediction based on fecal microbiota data links age with metabolic pathway regulation, such as that of the vitamin B_12_ synthesis pathway, which is predicted to decrease “age” and be associated with healthy aging [238]. Despite the indications that humans can absorb minute amounts of B_12_ independently of the intrinsic factor after an intrinsic factor system overload or when it has defects [239,240], increased intestine vitamin B_12_ levels may not provide significant amounts of B_12_ to the host due to the inefficiency of such passive diffusion (~1% of total vitamin B_12_ excess). Cobalamin receptors are located in the small intestine, while most of the vitamin B_12_-synthesizing bacteria reside downstream in the digestive tract in the colon [241]. Moreover, cobalamin constitutes only a tiny fraction of all corrinoids, at least in feces [242]. MMUT and MS can utilize cobalamin analogues for catalysis in vitro with a lower affinity and reaction rate; however, whether other corrinoids can reach MMUT in vivo for partial restoration of B_12_ deficiency is not known. Transfer of microbiota from young to old mice or from the wild type to prematurely aged mice prolongs their lifespan [243,244]. Moreover, oral supplementation with the *Akkermansia muciniphila* Muc^T^ bacterium only, which is more represented in feces of young mice, was sufficient for lifespan extension in prematurely aged mice. While *A. muciniphila* is not a vitamin B_12_ synthesizer, increased vitamin B_12_ concentration shifts its metabolism to decrease production of succinate and increase production of propionate and acetate [11]. However, some other species from the *Akkermansia* genus, including other *Akkermansia muciniphila* strains, are able to synthesize vitamin B_12_ de novo [11]. Interestingly, the *Akkermansia* genus as well as a B_12_-synthesizing *Bilophila* are also substantially increased in the microbiota of centenarians [245]. Increased cobalamin synthesis probably reshapes microbiota composition and metabolic pathways rather than being absorbed by the host [3]. Vitamin B_12_ tunes the gut microbiota, shifting the community towards the production of metabolites able to pass through cell membranes or helping to maintain the mucosal layer [3,243,246]. On the other hand, the microbiota, especially commensal Gram-negative bacteria, may accelerate immunosenescence in the colon. Overstimulation of B cells in the colon by lipopolysaccharide spawns their senescence, resulting in decreased amounts and diversity of microbiota shaping IgA [247]. Continuous exposure of macrophages to LPS may lead to the production of itocanate, which might deplete AdoCbl in nearby cells [231]. Some injuries, like colitis, an inflammation in the colon caused by altered microbiota, require partial dedifferentiation of cells for recovery, a process similar to cellular reprogramming. This process demands increased amounts of vitamin B_12_ because levels of holotranscobalamin in the serum decrease [179].

Thus, ingested vitamin B_12_ modulates microbiota in ways that are important for healthy aging.

## 7. Conclusions

In light of recent studies, vitamin B_12_ emerges as a modulator of aging. It is clear that deficiency in vitamin B_12_ disturbs cellular homeostasis by decreasing the capacity to fight oxidative stress, support of one-carbon and energy metabolism, and maintenance of proper epigenetic features (Figure 4). Metformin, initially an antidiabetic drug, is in clinical trials for use as an antiaging drug. It alleviates all hallmarks of aging [248] and regulates global DNA methylation through accumulation of SAM [249]. In a clinical study, metformin usage led to vitamin B_12_ deficiency [250], which may indicate an increased demand for vitamin B_12_ in cells. Scarcity of vitamin B_12_ exacerbates age-related cognitive decline, post-translational protein modifications, inflammaging, and reduced capacity for regeneration and potential for cellular reprogramming. High amounts of vitamin B_12_ are beneficial during gestation; however, they may have detrimental effects in elderly people due to the increasing probability of cancer and death, which fits the antagonistic pleiotropy theory of aging. During aging, transcription fidelity decreases, which may affect vitamin B_12_ ingestion and transport to cells [251,252]. Nonetheless, other possible mechanisms may also exist, such as deprivation of vitamin B_12_ caused by inflammaging, at least in macrophages. Adequate levels of vitamin B_12_ are needed for healthy aging, and antiaging interventions (i.e., cellular reprogramming) require an additional supply of B_12_. There is a need for standardization in determining vitamin B_12_ deficiency. We find it interesting to determine the role of B_12_ in senescence programs and its fundamental role in the implementation of epigenetic regulation programs, including SASP. Research studies on aging and age-related pathologies will benefit from methods allowing dynamic and specific assessments of cobalamins, including genetically encoded sensors with single-cell resolution, such as SenVitAL and its derivatives [253,254].

## Figures and Tables

**Table 1:** Functions of histone post-translational modifications.

Modification	Role	Reference
H3K9me2 H3K27me2 H3K9me3 H3K27me3 H3K79me3 H4K20me3 H2A/H4R3me2	Repression of gene expression	[83,84,90]
H3K4me1 H3K4me3 H2BK5me1 H3K9me1 H3K27me1 H4K20me1 H3K79me1 H3K79me2 H3K9ac H3K14ac H3K27ac H3K63ac H3K122ac	Increase in gene expression	[83,84,91,92,93,94]
H3K36me3	Prevention of transcription initiation outside promoters	[88]
H3K4 methylation	Prevention of DNA methylation	[89]

**Table 2:** Assessment of vitamin B_12_ in human blood plasma.

Analyte	Reference Interval	B_12_ Deficiency	Advantages and Disadvantages
Total B_12_, pM	200–600	<148	Requires digestion of proteins
Holotranscobalamin, pM	40–100	<35	Highly automated
Homocysteine, μM	8–15	>15	Several confounders: B_9_ and B_6_ levels, thyroid function, glomerular filtration rate, sex, and age
2-methylmalonic acid, nM	0.04–0.37	>0.37	Results may be unreliable in cases of kidney deficiency or infection
